# Accounting for spatial variation in climatic factors predicts spatial variations in mosquito abundance in the desert southwest

**DOI:** 10.21203/rs.3.rs-7595227/v1

**Published:** 2025-10-16

**Authors:** Kayode Oshinubi, Crystal M. Hepp, Ye Chen, Eck Doerry, Nicole Busser, John Townsend, James Will, Irene Ruberto, Melissa Kretschmer, Joseph Mihaljevic

**Affiliations:** Northern Arizona University; Northern Arizona University; Northern Arizona University; Northern Arizona University; Vector Control Division; Vector Control Division; Vector Control Division; Arizona Department of Health Services; Maricopa County Department of Public Health; Northern Arizona University

**Keywords:** West Nile virus, Culex, Clustering, Mosquitoes, Climate

## Abstract

**Background:**

Mosquitoes are vectors for diseases globally, making development of models that better explain mosquito abundances imperative. Mosquito population dynamics are particularly sensitive to local weather conditions, and mosquito-borne disease outbreaks can be spatially concentrated. There is a need for improved modeling studies to address whether spatial variation in disease outbreaks is driven by spatial variation in weather conditions, especially in dry and hot environments. In this study, we build a climate-driven model of mosquito population dynamics and compare whether predictions of mosquito abundance at the county scale are improved by accounting for sub-county climate variation.

**Methods:**

Using a 5-year time series of weekly mosquito abundance data collected for each zip code in Maricopa County, USA, we assess how local variation in climate can explain and predict mosquito population dynamics. We built a mechanistic model of mosquito population dynamics influenced by daily temperature and 30-day accumulated precipitation. We grouped zip codes based on similar patterns of temperature and precipitation using functional clustering. We compared two approaches: one using county-level average climate and another using data from the identified climate clusters. We use MCMC to fit the mechanistic model using averaged climate data in each cluster, then compare the modeling fit to observed data of the county-level model to the model based on climate-based clusters.

**Results:**

Simple, climate-forced modeling accurately estimates detailed mosquito abundance trajectories throughout a five-year period. Modeling mosquito abundances in the sub-county spatial clusters demonstrated that the same effects of temperature and precipitation on population growth rates could explain small-scale changes in mosquito populations. However, when we aggregate the sub-county model fits to the county-scale, the resulting fits are more precise but are sometimes overly confident, leading to lower overall accuracy and predictive performance.

**Conclusions:**

Our study demonstrates the importance of collecting fine-scale mosquito abundance data to improve our understanding and the predictability of mosquito population dynamics. The strong performance of both the cluster-based and county-level models illustrates the value of spatially sensitive modeling in this application. We anticipate that such modeling efforts will also aid in using weather forecasts to predict mosquito populations, aiding in efforts to control the spread of infectious disease.

## Background

Mosquitoes are carriers of many pathogens that have global impacts, including West Nile virus (WNV), Yellow Fever Virus, Rift Valley Virus, Zika Virus, Chikungunya Virus, Dengue, and Malaria, which cause more than 700,000 deaths annually [1]. For example, as of 2022, 249 million cases of malaria were identified worldwide [2]. Dengue virus is widespread in over 100 countries, with annual global infections of 390 million persons [3]. Furthermore, one in every 150 people infected with WNV is diagnosed with neuroinvasive WNV disease, with many suffering from long-lasting secondary sequelae (e.g., depression, memory loss, and motor dysfunction). As ectotherms and complex-life-cycle organisms, mosquito population dynamics are invariably linked to climate and local environmental conditions [4, 5], such as temperature, precipitation, humidity, and the availability of standing water for breeding. It is well documented from laboratory studies that temperature drives the rates of many key demographic traits across different mosquito life stages and across many mosquito species [6]. Interestingly, humidity can alter the shapes of these thermal performance relationships, indicating that interactions between climate factors will also impact population dynamics [7]. For some mosquito species, the availability of standing water for breeding helps explain trends in population sizes, such that cumulative measures of precipitation are often used as proxies to model mosquito populations. A recent study in [8] analyzed the relationships between mosquito population size, irrigation, and WNV cases in humans, finding an association between mosquito population and irrigation activities. Accordingly, climate factors show statistical relationships with the burdens of many mosquito-borne diseases. For instance, statistical modeling studies often use climate factors to explain and predict the cases of WNV in humans [9–14]. Based on the relationships between climate, mosquito population demography, and disease burden, more recent studies attempt to project the effects of climate change on the future global distributions of mosquitoes and the diseases they harbor [15–19]. Given the complicated nature of climate’s effects on mosquito population dynamics, flexible modeling approaches are needed to more mechanistically explain how multiple climate variables influence variation in mosquito abundance patterns over space and time.

Mosquito population dynamics can fluctuate at relatively small spatial scales, likely because local weather variation leads to spatial and temporal variation in mosquito abundances [20–22]. For instance, even across a few hundred squared kilometers, mosquito population sizes can vary spatially and seasonally, potentially due to differences in local precipitation and temperature, and the effects of weather may even vary among co-occurring mosquito species [23]. An important challenge is therefore not only to build mechanistic models that can explain temporal trends in mosquito population fluctuations, but that also help us interrogate the factors that explain spatial heterogeneities in these abundance patterns.

Many methods are used to model the spatial and temporal fluctuations in mosquito population abundances, ranging from purely mathematical models (e.g., compartmental differential equation models) to purely statistical models (e.g., linear regression frameworks). Developing an adequate mathematical or statistical model to explain or predict mosquito population dynamics in relation to climate is a challenging endeavor with various trade-offs. Purely statistical models, including statistically-based machine learning algorithms, can do a good job of explaining temporal and spatial trends in mosquito abundances [8, 9, 24–28]. A challenge with statistical modeling approaches is that they typically are inadequate representations of the well-known non-linear effects of climate on complex population dynamics. Additionally statistical models are generally correlational, meaning that at least some mechanistic understanding of the system is lost. Classic [29–31] and contemporary mathematical models often introduce large sets of differential equations that represent the various life-stages of mosquitoes, from egg to larvae to adult, and they can include the effects of temperature on certain demographic rates [32–36]. Such models have the strong benefit of inherently including non-linear effects of climate on demographic patterns, but they also have some challenges. It is difficult to derive realistic estimates for the many parameters in such large models (i.e., a dimensionality problem). Parameterizing such models therefore often relies on laboratory experiments that measure thermal performance of mosquitoes, but such parameter estimates are largely untested in field settings (although see [37]). Fortunately, recent mechanistic modeling and model-fitting approaches show promise in incorporating both temperature and precipitation to explain dynamic mosquito population abundance patterns [15].

In our study, we seek to develop a mechanistic yet simplified way of incorporating temperature and precipitation into a generic and dynamic modeling approach. This modeling approach allows us to explore if spatiotemporal variation in weather explains the non-linear dynamics of mosquito populations in natural landscapes. We test our modeling approach using the study system of *Cx. quinquefasciatus* in Maricopa County, AZ, which transmits multiple infections to humans, and is the most abundant vector for West Nile and St. Louis Encephalitis (SLEV) viruses in the county. According to the CDC, Maricopa County is consistently in the top 10 counties yearly in the nation for West Nile virus cases [1]. From 1999 to 2022, Maricopa County has had the most human cases in the nation, with a total of 3006 infections. Hundreds of traps are set weekly across the county, and mosquito abundances can vary dramatically in magnitude across subdivisions of the county. Hence, this system is ideal for testing how a combination of weather factors may influence spatiotemporal heterogeneity in mosquito population dynamics. We therefore hypothesized that accounting for microclimate variation—by grouping areas with similar weather patterns—would improve our model’s ability to predict mosquito abundance across the county. This modeling study is important because it helps us understand how spatial variation in climate affects spatial variation in mosquito population sizes. Moreover, in future work, the simplified modeling approach we develop here could be incorporated into epidemiological models to account for important climate and spatial variation in disease transmission.

## Methods

### Approach Overview

First, we develop a semi-mechanistic model of the temporal dynamics of adult mosquito abundance, which incorporates non-linear statistical effects of temperature and accumulated precipitation. We then use Bayesian Markov chain Monte Carlo (MCMC) to fit our model to three years of weekly, adult female *Cx. quinquefasciatus* abundance observations across Maricopa County (i.e., the abundance data aggregated across all traps established in the county). To test whether spatial heterogeneity in climate explains variation in the patterns of mosquito abundance across Maricopa County, we cluster the county’s zip codes by local similarities in climate, and aggregate the mosquito abundance across traps within these clusters. We develop a method to hierarchically re-fit our model to abundance data in each cluster. Next, we compare whether fitting the data per cluster and then aggregating these fits to the county-level improves the overall fit of the county-level data. In this way, we see if heterogeneity in climate-based spatial clusters can help us better explain the county-level aggregated data. Finally, we also evaluate the out-of-sample prediction accuracy of our models, at both county and cluster scales. Below, we describe each step of the process in detail.

### Climate-forced mathematical model

We developed a mathematical model that explains temporal patterns of adult mosquito abundance, which is driven by two exogenous climatic variables: temperature and precipitation. The general form of the ordinary differential equation is

$$\frac{dX(t)}{dt}=\nu \;f(Temp(t),Prcp(t))-\mu X(t).$$

X(t) is the mosquito abundance over time, with model time steps discretized to daily as $X\left(t_{i}\right)$ where i=1,2,3,…. The model assumes that there is a baseline population growth rate of mosquitoes, ν, which is modulated by a statistical function of daily measures of temperature Temp(t) and precipitation Prcp(t). Specifically, Temp(t) is the daily maximum temperature in degrees Celsius, and Prcp(t) is the daily measure of 30-day accumulated millimeters of precipitation, which we scale for statistical convenience by dividing by a constant 50.

The form of the statistical function is:

$$f(Temp(t),Prcp(t))=-(Temp(t)-Tmin)(Temp(t)-Tmax))\frac{1}{\left(1\;+\;e^{\alpha -\varphi \;Prcp(t)}\right)}$$

The temperature effect is modeled as a quadratic equation, representing a thermal performance curve of population growth rate, controlled by a minimum Tmin and a maximum Tmax growth temperature. This structure is agnostic to the fact that many traits of mosquito individuals across their life stages have unique thermal performance curves [6]. Our strategy is instead to test a simplification by estimating a parsimonious representation of thermal effects on the overall growth rate of the adult mosquito population, integrating across the thermal effects of specific, life-stage specific traits. The precipitation effect is modeled as a logistic, saturating function, whose shape is controlled by constants α and φ. Biologically, this means that as accumulated precipitation increases, the growth rate increases, but the model could estimate how quickly this increase occurs across the range of precipitation and whether there is a saturating effect, above which more accumulated precipitation has negligible effects. Illustrative plots of the temperature and precipitation functions and their corresponding seasonal time series are shown in Additional file 1: Fig. S1.

We fix the per-capita mortality rate μ (at 0.12) instead of estimating it as the ratio between the growth term and the mortality term are the only factors that govern the net population change; estimating both would create an identifiability issue. Consequently, fluctuations in the fitted growth term implicitly capture both births and deaths.

In Table 1, we described each parameter in our mathematical model.

### Mosquito abundance data

The mosquito abundance data used in this article was collected by the Maricopa County Environmental Services Vector Control Division [38]. The data is collected weekly from ~ 800 CO_2_ traps located in an approximate 1km ^2^ grid across most of the county. The longitude and latitude of the traps locations is documented for easy spatial analysis. Each trap is placed for a 12-h collection period once per week for 50 weeks of the year. Collections are subsequently sorted by mosquito species and sex. Data used in this study were restricted to *Cx. quiquefasciatus*, the most frequently trapped mosquito vector in Maricopa County, and is known to carry avian malaria, WNV, SLEV, and other pathogens. This species constitutes about 80% of the total infected mosquitoes in Maricopa County.

**Figure 1:**
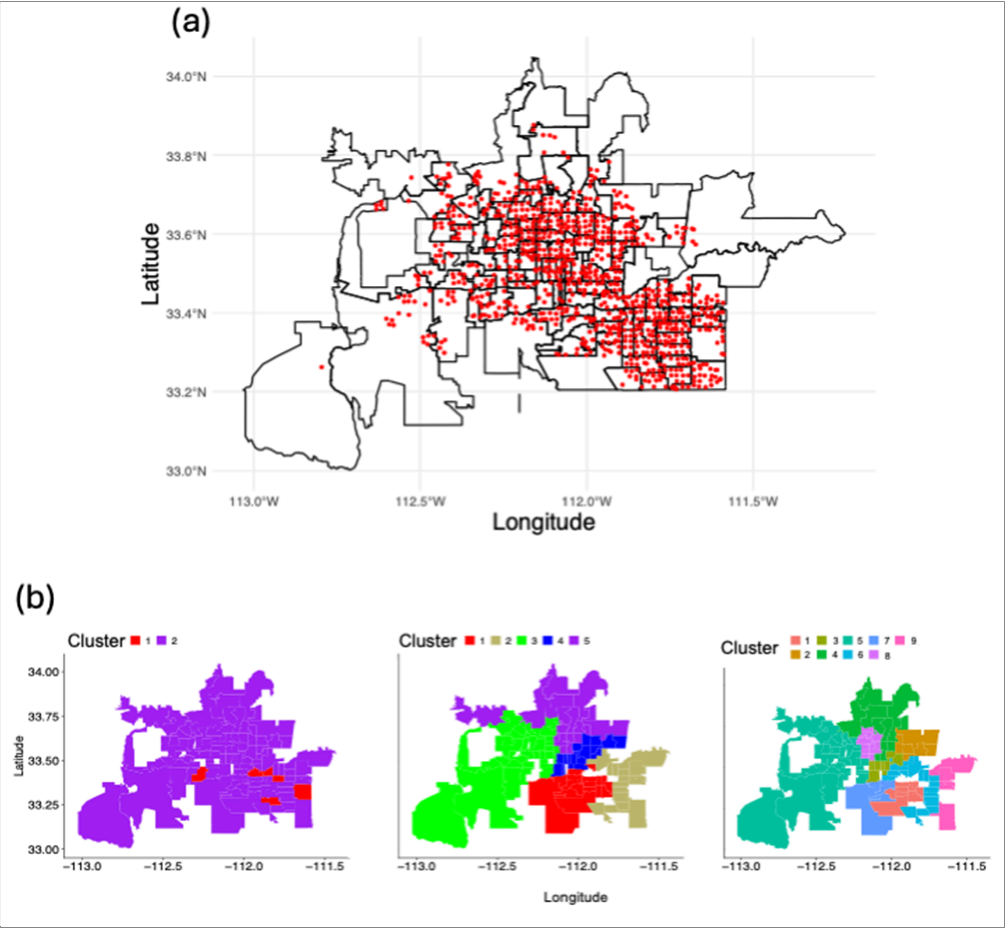
**(a)** Map of mosquito trap locations and ZIP Code Tabulation Areas (ZCTAs) in Maricopa County. Each point represents a CO_2_-baited mosquito trap monitored weekly by the Maricopa County Environmental Services Vector Control Division. Traps are spaced approximately 1 km^2^ apart and record species- and sex-specific mosquito counts during 12-hour collections. For this study, we aggregated adult female Cx. quinquefasciatus counts at the ZCTA level to analyze spatial patterns in mosquito abundance and perform climate-based clustering of ZCTAs for model training and inference. **(b)** Clustering result. 2 clusters represent six outlier ZCTAs (red) versus all others (purple), whereas the 5 and 9 clusters were clustered based on precipitation data of all other ZCTAs.

In our analysis, we used a training data set spanning three years, January 1, 2014 to December 31, 2016, and we used 2013 and 2017 data to validate our model as an out-of-sample prediction data set. We chose 2014–2016 for the training set, because the data includes unique patterns in abundance that can help validate the effects of climate. For instance, in 2014 there was a large monsoon season, and this effect can be visually observed in the abundance data, while years 2015 and 2016 had relatively average abundance trends. For the county-level data set, mosquito abundance per week was aggregated across all traps. We used data for adult female mosquitoes only since they were blood-seeking and attracted to CO_2_ traps. Each trap is identified by GPS coordinates (longitude and latitude), such that for the cluster-level data sets, we first aggregated trap data to the ZIP code-level (i.e., ZIP Code Tabulation Areas, ZCTA), and then clustered ZCTA by climate similarity and aggregated trap data accordingly (see Fig. 1a).

### Temperature and precipitation data

**Figure 2:**
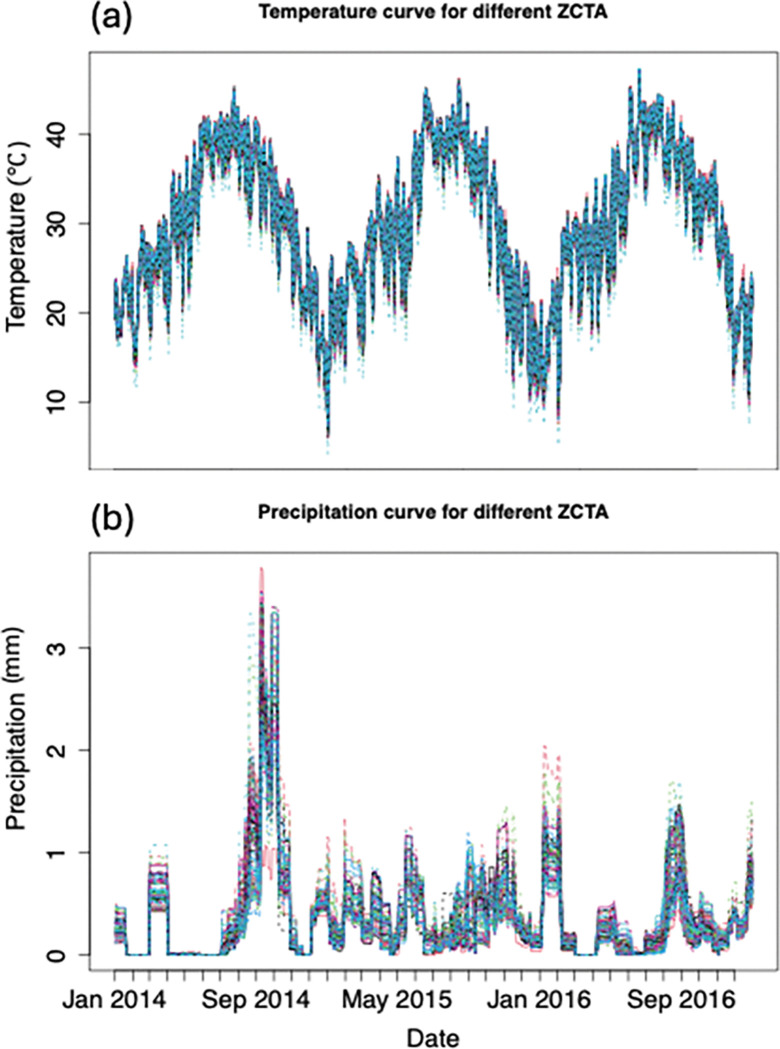
Daily climate variables in Maricopa County from 2014 to 2016. **(a)** Daily maximum temperature (°C) and **(b)** daily precipitation (mm), both averaged across all ZCTAs within Maricopa County using 4 km × 4 km PRISM gridded data. These aggregated time series were used as climate inputs for both county- and cluster-level mosquito abundance models. The different colors represent temperature and precipitation for each ZCTA.

Daily estimates of maximum temperature in Celsius and precipitation in millimeters were downloaded from PRISM via the prism R package [39]. We downloaded the Prism daily data in a 4 km by 4 km raster. For the county-level model, we averaged daily values across all the Prism data in the raster within the Maricopa County polygon defined by the 2010 Census Bureau delineations (tigris package [40]) in Maricopa County. For the cluster-level model, we averaged the Prism data across the grid within each ZCTA polygon, again using 2010 Census Bureau delineations. Then, we averaged these data across all ZCTAs included in a given cluster. In the model, we used a 30-day accumulated precipitation, scaled by dividing by the constant 50. In Fig. 2, we present the time series plot for temperature and precipitation for 2014–2016 which is averaged across ZCTA in Maricopa County.

We explored the spatial distribution of the mosquito abundance data present within the 109 non-outlier ZCTAs across Maricopa County (see details of outlier ZCTAs in Additional file 1: Text S1). Our goal was to cluster these ZCTAs based on similarities in climatic data during the three-year window of the training data set. We used only the precipitation time-series data, as the temperature time-series did not vary significantly across the county (see Fig. 2). Using the precipitation data, we clustered the ZCTAs based on a functional time series clustering technique implemented in the FunFEM package in R [41]. This clustering technique requires the user to specify the number of clusters desired. Therefore, we tested 5 and 10 clusters. Briefly, to apply functional time-series clustering, first, a basis function must be fit to the time-series data to smooth it. In our case, we used a Fourier basis, which is the most appropriate for periodic patterns, such as those observed in the precipitation data [42].

The functional clustering algorithm compares time-series data by transforming them into functional forms, often using basis expansions like splines or Fourier series, to capture their continuous-time dynamics [42]. It then measures similarity based on shared features such as trends, seasonal patterns, or amplitude variations using a model-based clustering approach in a discriminative functional subspace, where differences between curves are captured via latent variable modeling and Gaussian mixture models, primarily relying on functional Principal Component Analysis (PCA) and Fisher-like discriminative analysis. Time series with similar functional properties are grouped into contiguous clusters, allowing for the identification of common behaviors and trends across the data while accounting for variability in shape, scale, and temporal alignment.

Note that when we specified 10 clusters, one cluster only included four ZCTA, each with a maximum mosquito abundance per week of less than ten. Therefore, we manually moved those to the most similar other cluster, leaving us with a comparison of 5 versus 9 clusters (see middle and last panel of Fig. 1b).

### Fitting the model to the time-series data

We used a Metropolis-Hastings Markov chain Monte Carlo (MCMC) algorithm to sample from the joint posterior distribution of model parameters. To calculate the likelihood, we compare the solution of our ordinary differential equation model to the weekly observed abundance data. We solve the differential equation model with the deSolve [43] package using the method lsoda [44] in R, incorporating daily fluctuations in temperature and precipitation. We compare the model to the data every seven days; therefore, while the model ingests daily data on climate, the model is only compared to the observed mosquito data once per week. The likelihood is defined as a negative binomial probability distribution with an estimated over-dispersion parameter (see Additional file 1: Text S2-S3 for more details). For each spatial data set, we ran four MCMC chains, each with 5,000 iterations, in parallel using the futures R package [45]. We used the $\hat{R}$ statistic [46], also known as the Gelman-Rubin diagnostic, to assess the convergence of the chains. We also performed posterior predictive checks, described below, to ensure the joint posterior defined a reasonable parameter estimation space.

We fit the model to the following 5 data partitions: county-level with outliers, county-level without outliers, 2-cluster (outlier ZCTA versus all other ZCTA), 5-cluster without outliers, and 9-cluster without outliers. For the two county-level partitions, we run a standard MCMC process. For the cluster-level partitions, we ran a hierarchical inference approach.

### Model validation

We used within-sample and out-of-sample predictive measures to compare model performance across the different data partitions. We are ultimately interested in understanding if accounting for spatial variation in climate and baseline growth rates might improve the model’s explanation of county-wide, total mosquito abundances. Therefore, in calculating goodness-of-fit metrics, for the cluster-level data partitions, we summed metrics across clusters to a county-level performance metric.

The method we employed for goodness-of-fit measurement was Root Mean Square Error (RMSE). To generate this measurement for each data partition and model-fit, we first generate posterior predictive model runs. For each data partition and MCMC analysis, we randomly sampled 100 parameter sets from the joint posterior. For each set, we substitute the ODE to generate the corresponding numerical solution. Across these 100 model runs, we calculated the median model expectation per day for the model. The RMSE was then calculated by comparing the data observations to the median model expectation, and summing across all observations, and across clusters, when appropriate.

## Results

### Variation in precipitation and temperature

**Figure 3:**
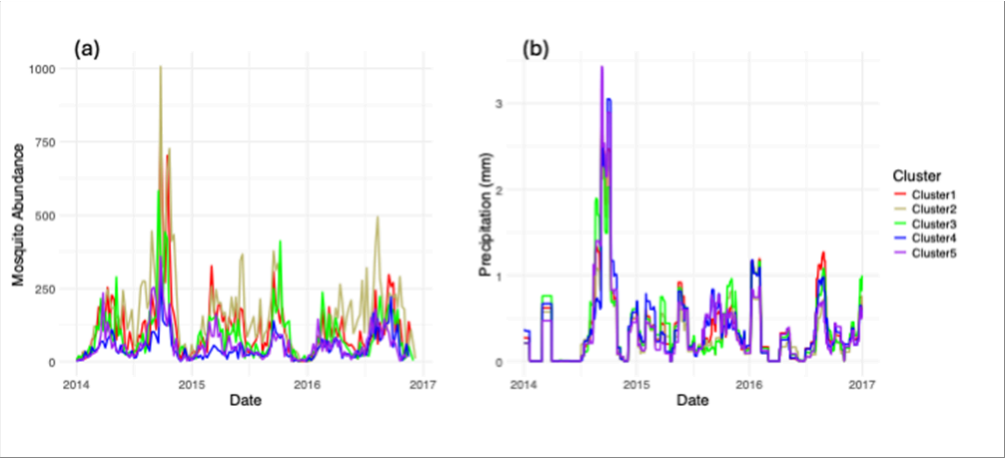
Spatio-temporal patterns in precipitation and mosquito abundance across five climate-based clusters of ZCTAs in Maricopa County. **(a)** 30-day accumulated precipitation (mm), showing differences in precipitation magnitude and timing among clusters. **(b)**Weekly mosquito abundance, highlighting cluster-level variation in response to climatic conditions, particularly during the 2014 monsoon season and mid-to-late 2015.

The clustering routine captured logical spatial groupings of zip codes (ZCTA) across Maricopa County based on spatial and temporal variation in 30-day accumulated precipitation (Fig. 2 and Fig. 3). When we look at the PRISM-derived precipitation metric, we see that clusters are mostly differentiated by precipitation magnitude and more minimally by precipitation timing. For instance, in the 5-cluster case, all clusters experienced relatively high abundance in the monsoon season of 2014, with minor variation in total magnitude, whereas in 2015, there is some variability in the timing of precipitation, especially in the second half of the year (see Fig. 3).

### Comparing county- and cluster-level modeling

**Figure 4:**
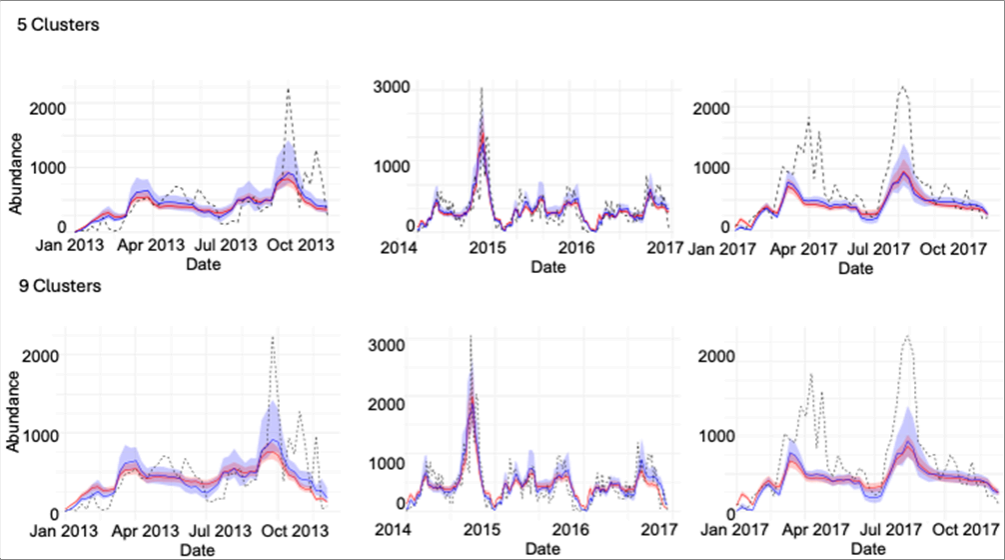
Comparison of clustering prediction, real data, and county prediction for 5 (top) and 9 (bottom) clusters. Shaded ribbons in blue indicate 95% credible intervals for county prediction, while shaded ribbons in red indicate 95% credible intervals for cluster sum prediction. The black dotted lines represent observed mosquito abundance data over time.

In general, our mechanistic model captures detailed patterns of mosquito abundance changing through time across multiple spatial scales for the three years of training data, particularly when we remove the six outlier ZCTA (Fig. 4 and Additional file 1: Fig. S6-S7). For example, the model generally captures the effects of high summer temperatures, where we see corresponding declines in mosquito abundances, and also the effects of monsoonal activity, where we can see dramatic increases in mosquito abundances due to heavy rainfall (e.g., in the second half of 2014).

Accounting for heterogeneity in precipitation across clustered ZCTA lead to higher precision in model fits we aggregated model expectations from cluster to county-scales (i.e., narrower error bands); however, these aggregated model fits were sometimes overly confident, such that the error bands did not always capture the true observations (see Fig. 4). Thus, the county-level model, which used daily values for temperature and precipitation that were averaged across the ZCTA, had a relatively better fit compared to the aggregated cluster-level models, based on the Root Mean Squared Error (RMSE) metric (Additional file 1: Table S3-S4). Furthermore, there were not clear differences in the aggregated county-level model fit when we compare the 5-cluster to the 9-cluster situations (Fig. 4). When we examine model fits per cluster, however, we do see that the model captures variation in mosquito abundances across clusters, explained mostly by differences in baseline population growth rates (Additional file 1: Fig. S6-S7). Also, we observed that the RMSE values vary across clusters depending on the number of clusters (Additional file 1: Table S3-S4). This result demonstrates that our method of hierarchical estimation of cluster-specific growth rates successfully characterized key differences among clusters. Collectively, the results suggest that the aggregate, county-level patterns in mosquito abundance can be most parsimoniously explained by a spatial average of temperature and precipitation data. Yet the cluster-level models provide accurate, finer-scale inference of how mosquito population varies across space and time, though at this finer scale the model fits may be more prone to small prediction errors. Additional details about outliers and county level analysis can be found in Additional file 1: Text S4-S5.

### Out of sample performance

When we predicted mosquito abundance for two additional years of withheld data (2013 and 2017), our model still captures the fundamental characteristics of these mosquito abundance time-series (see Additional file 1: Fig. S6 (first and last panel), S8-S9). Indeed, the key results from the training data still hold true with the out-of-sample data. Particularly, the county-level model performs nearly equally as well as the cluster-level models. Notably, however, across spatial scales, the dynamic model does a poor job at explaining the early-year increases in mosquito abundance observed in 2017, and under-predicts peaks in abundances observed in the later half of both 2013 and 2017. We observed these failures of the model in both the county- and Cluster-level out-of-sample model fits.

## Discussion

This research sheds light on the complex relationships between climate, spatial heterogeneities, and mosquito populations. By implementing a novel mechanistic model with a data-driven clustering strategy, we show that a relatively simple mathematical model that incorporates temperature and precipitation effectively captures the dynamical patterns of mosquito abundance observed over multiple years in a large urban setting. When we fit our model to aggregate data for all of Maricopa County, our climate-driven model accurately explains the seasonal increase in mosquito abundances in the spring, a decline in the hottest part of the summer, and another increase in the warm, wet monsoon season. When we subdivided the county into clusters based on similar climate patterns, we observe that average mosquito abundances vary substantially across the county. However, the cluster-level model still explains a substantial proportion of variance in the these downscaled data, indicating that mosquito populations across the region exhibit consistent numerical responses to temperature and precipitation. When we aggregate these smaller-scale model fits, we improve precision around the median model prediction, but we do not improve county-level model accuracy—likely due to error accumulation across clusters and the sparse data within each group. What this tells us is that the same climate-forced model can be applied at sub-county spatial scales and will explain the key dynamical patterns of the mosquito population dynamics; yet, at these smaller spatial scales, the model will be more prone to observational error, suggesting a trade-off between spatial resolution and model accuracy. Our results underscore the importance of modeling spatial structure when interpreting mosquito dynamics, particularly in regions like the desert Southwest where precipitation and temperature vary dramatically over small spatial scales.

Previous studies have demonstrated that local weather conditions influence mosquito abundance and distribution [15, 20, 47, 48]. Research on *Cx. quinquefasciatus*, the focal species in our study, has highlighted how temperature and precipitation shape its life cycle. For example, a study in Hawaii [49] found that *Cx. quinquefasciatus* abundance increased with temperature, peaking at mid-elevation sites, and showed a non-linear relationship with precipitation—highlighting temperature as a key driver of mosquito distribution across varying terrains. Precipitation also played a significant, albeit complex, role. Interestingly, the relationship between precipitation and abundance indicated negative effects of rainfall, while lagged precipitation showed positive associations. This complexity likely reflects seasonal rainfall patterns and their influence on larval habitat availability. Similarly, Morin and Comrie [50] used the Dynamic Mosquito Simulation Model (DyMSiM) model to show that the interaction between temperature and precipitation drives mosquito dynamics differently in California and Florida. Valdez et al. [51] further revealed that not only total rainfall, but also the number of rainy days and daily variability, strongly influence mosquito abundance. Our findings corroborate these earlier studies and extend them by explicitly testing whether accounting for spatial climate heterogeneity improves the performance of a mechanistic model.

Although the cluster-level model explains key features of the mosquito population dynamics at smaller spatial scales, aggregating cluster-level predictions to the county scale did not improve inference. A possible contributing reason for this contradiction is that the data we used per mosquito trap (PRISM), interpolates weather patterns across space based on sparse station networks and provides 4×4km grid raster data. In Arizona, localized monsoon events produce intense, short-duration rainfall that varies significantly over short distances. PRISM’s data resolution may smooth over these extremes, missing key signals needed for accurate prediction [52, 53]. Future studies could consider deploying ground-based weather stations near the mosquito traps to obtain high-resolution climate data. This could enhance the accuracy of fine-scale model predictions. Moreover, model error may accumulate during the aggregation process, particularly when fit to clusters with small sample sizes. While the cluster-aggregated model did not improve county-wide predictions, it remains valuable for local inference and public health planning. Local-level fits can inform targeted mosquito control strategies and enhance situational awareness.

In our framework, we estimated a shared climate-response function for the mosquito population growth rate across all the clusters – the quadratic effect of temperature and the exponentially saturating effect of accumulated precipitation. Given that this same functional response explains the data across clusters, this provides evidence that mosquito sub-population growth rates are responding similarly to weather despite differing average abundances. However, the current approach does not account for mechanisms that may explain the average differences abundance across clusters. Future research should explore how landscape features, water availability, or urban infrastructure might explain baseline variation in mosquito abundances.

We also encountered six zip codes with unusually high mosquito counts, especially in spring, which the model failed to capture even when fit separately. These outlier patterns were not explained by temperature or 30-day precipitation trends, suggesting local landscape effects such as standing water accumulation may be at play. Investigating these features—e.g., water-retaining infrastructure, wetlands, or irrigation systems—could yield important insights. Future work could incorporate additional covariates to explain baseline mosquito growth rates, such as proxies for standing water, land use, and hydrological features. High-resolution, daily updated data on these variables could better capture early-season surges in abundance. Additionally, spatial movement of mosquitoes—whether natural or human-facilitated—should be considered in spatially explicit models.

Spatial processes are known to structure ecological and epidemiological dynamics, influencing everything from vector distribution to disease transmission and species interactions [47, 54]. Invasive species spread, habitat fragmentation, and local resource competition all highlight the importance of spatial structure in population persistence and disease risk [55]. By explicitly incorporating spatial heterogeneity, our model represents a step forward in mosquito modeling efforts. As weather forecasting capabilities continue to improve, spatially structured models will be critical for translating environmental changes into actionable public health responses.

Climate change is reshaping ecosystems and disease risk globally [16–18]. Rising temperatures and altered precipitation patterns create more favorable conditions for mosquito proliferation and disease transmission [56, 57]. Accurate models that incorporate both temporal and spatial heterogeneity are essential to predict these dynamics and guide interventions.

## Conclusions

In sum, our study demonstrates the value of spatially resolved climate-driven models for understanding mosquito population dynamics. Although challenges remain in scaling predictions and capturing outlier behavior, this work lays a foundation for more refined, localized modeling efforts that can enhance mosquito control and public health preparedness in a changing climate.

## Supplementary Material

Supplementary Files

This is a list of supplementary files associated with this preprint. Click to download.

• MosqAbundsupp.pdf

• abstactfigure.tiff

## Figures and Tables

**Table 1:** Parameters of the model

Parameter	Signification of parameters	Unit	Source
*ν*	Baseline population growth rate	$\frac{\text{mosquitoes}}{{}^{\circ}C\cdot \;\text{Day}}$	Estimated
*Tmin*	Minimum temperature that constrains population growth	° *C*	Estimated
*Tmax*	Maximum temperature that constrains population growth	° *C*	Estimated
*α*	Contributes to the inflection point of the precipitation effect curve	Unitless	Estimated
*φ*	Steepness of the precipitation effect curve	1/mm	Estimated
*μ*	Mosquito death rate	1/day	Fixed

**Table 2:** Model evaluation comparison at the county level

Model	Fitted RMSE (2014–2016)	Predicted RMSE (2013)	Predicted RMSE (2017)
County without ‘outliers’ ZCTAs	252.6900	306.0828	494.7659
5 clusters sum	265.8615	325.5769	507.0085
9 clusters sum	269.4774	349.3141	511.3981

## Code availability

Code used for analyzing the data for this research is available upon request.
